# Distribution and associated factors of neck and shoulder pain among primary school children in Shijiazhuang city, Hebei province, China

**DOI:** 10.3389/fpain.2026.1822093

**Published:** 2026-05-29

**Authors:** Sha Li, Eva Nabiha Zamri, Purwo Sri Rejeki, Xue Yi

**Affiliations:** 1 Department of Community Health, Pusat Kanser Tun Abdullah Ahmad Badawi, Universiti Sains Malaysia, Pulau Pinang, Malaysia; 2 Physiology Division, Department of Medical Physiology and Biochemistry, Faculty of Medicine, Universitas Airlangga, Surabaya, Indonesia; 3 Department of Sports Science, Hebei Sport University, Shijiazhuang, China

**Keywords:** musculoskeletal pain, neck pain, physical activity, prevalence, primary school children, shoulder pain

## Abstract

**Background:**

Neck and/or shoulder pain (NSP) is widely reported among school-aged children, with documented detrimental effects on their physical health and psychological status. This study aims to identify the prevalence and associated factors of NSP among primary school children in Shijiazhuang City, Hebei Province, China.

**Methods:**

In this cross-sectional study, 362 primary school children aged 10–12 years from Shijiazhuang city, northern China, were recruited using a multistage cluster random sampling method. Participants completed the fundamental motor skills test and questionnaires assessing socio-demographics, physical activity, psychological distress, quality of life, and NSP. Data were analyzed using univariable and multivariable logistic regression.

**Results:**

The one-month prevalence of NSP was 33.7%. Multivariable logistic regression showed that female students had a significantly higher odds of NSP compared with males [adjusted odds ratio [AOR] = 1.61, 95% confidence interval [CI]: 1.01–2.56, *P* = 0.047]. In addition, higher psychological symptom scores were independently associated with an increased odds of NSP (AOR = 1.04, 95% CI: 1.01–1.09, *P* = 0.036). Conversely, moderate physical activity (AOR = 0.51, 95% CI: 0.29–0.89, *P* = 0.018) and higher quality of life scores (AOR=0.98, 95% CI: 0.95–0.99, *P* = 0.026) were linked to lower odds of NSP.

**Conclusion:**

NSP is prevalent among Chinese children. Moderate-intensity physical activity, quality of life, and psychological status were associated with NSP. These findings highlight the importance of implementing early interventions that promote physical activity and psychological well-being to mitigate NSP in children.

## 1 Introduction

Musculoskeletal pain (MSP) in children has emerged as a growing public health concern (1). It has been estimated that up to 40% of children experience MSP (2). Neck and shoulder pain (NSP) has become one of the most persistent MSP symptoms among primary school children (3). Prior research has indicated that the prevalence of NSP in children ranges from 20% to 40%, with rates increasing with age (4, 5). Moreover, evidence further suggests that NSP may be more prevalent among children and adolescents in developing countries (6–8). A study conducted in Iran reported a prevalence of 32.3% among primary school students (6). Similarly, research from Brazil identified a prevalence of 30.1% among children aged 10–14 years (8). These findings indicate that NSP exhibits a relatively high prevalence during childhood.

Longitudinal evidence indicates that NSP often persists across childhood and adolescence (4, 9). For example, NSP is already relatively common at the age of 10 (approximately 20%) and increases to around 30% by age 15 (4). Importantly, childhood pain has also been identified as a potential risk factor for negative health outcomes in adulthood (10–12). It has been reported that in a six-year longitudinal follow-up study, approximately 72% of adolescents reported persistent pain in young adulthood (12). In adults, neck pain prevalence has been reported to reach nearly 86.8% (13), substantially impairing health and quality of life (11, 12). Therefore, assessing NSP in children may help elucidate related conditions that could emerge later in life. To mitigate the adverse consequences of NSP, early prevention is essential.

Previous studies have established evidence on the contributing factors of NSP among adults (14–16). Nonetheless, the prevalence of NSP and its associated factors among primary school children remain under-investigated. Existing studies have examined risk factors for NSP among children, including psychological factors (e.g., anxiety and depression) (4, 17), school-related factors (e.g., desk and chair height and excessive homework) (5, 6), and lifestyle factors (e.g., poor sitting posture and physical activity) (6, 7, 18). However, findings on the associations between these factors and NSP remain inconsistent. Specifically, some studies have found no significant association between physical activity and NSP (3, 19), whereas others suggest that higher levels of physical activity are associated with an increased risk of NSP (20), while moderate levels appear to be protective (18, 21, 22).

In China, due to the heavy academic burden, many students do not engage in sufficient physical activity (23–25), and the increasing reliance on electronic devices in both learning and daily life may further exacerbate this trend (26–29). Particularly in the post-COVID-19 context, increased dependence on digital devices and reduced time spent in outdoor activities may have altered students’ learning patterns and daily behaviors, potentially influencing the occurrence of NSP (7, 30, 31). However, research focusing on the prevalence of NSP and its associated factors among Chinese primary school children remains limited. Therefore, this study aims to identify the NSP prevalence and associated factors among Chinese primary school children.

## 2 Methods

### 2.1 Study design, study setting, and sampling method

A cross-sectional study was conducted in the primary schools located in the four urban districts of Shijiazhuang City, Hebei Province, China. There were four urban districts (Xinhua, Yuhua, Qiaoxi, and Chang'an) and three suburban districts (Luquan, Luancheng, and Gaocheng) in Shijiazhuang City. We selected urban locations because (i) urban children are more exposed to academic pressure, prolonged sitting, and screen use (32), and (ii) focusing on urban districts reduced heterogeneity in school resources and environments, thereby improving the internal validity of this.

We adopted a multistage cluster random sampling method to select schools and classes, followed by the recruitment of student participants. First, within each district, approximately 50% of primary schools were selected using simple random sampling, from which two schools were randomly chosen. Second, in each selected school, one class was randomly selected as the cluster. Finally, all eligible students in the selected class were invited to take part in the study.

### 2.2 Study participants

We recruited primary school students aged 10–12 years who were able to complete testing and questionnaires and provided assent, with parental consent and school permission. The screening process was based on health reports provided by parents and physical examination records of the students (school health records). Exclusion criteria were serious heart or respiratory diseases or other serious physical illnesses, severe cognitive dysfunction, chronic MSP caused by infectious diseases or other juvenile inflammatory conditions, and a history of congenital or acquired serious bone diseases.

### 2.3 Sample size calculation

Sample size was calculated using OpenEpi (version 3.01), an open-source epidemiologic calculator (33). A prior study reported an NSP prevalence of 32.3% among primary school children (6). Using this estimate and applying a 95% confidence level and a 5% absolute precision results in a required sample size of 336. Considering an anticipated 10% non-response rate, the target sample size was set at 370 participants.

### 2.4 Study measures

#### 2.4.1 Neck and/or shoulder pain (NSP)

The Nordic Musculoskeletal Questionnaire (NMQ) was employed to assess MSP in multiple body regions (34). Participants were asked whether they had experienced pain during the past month and during the past six months. Two items from the NMQ assessed pain: “In the past month, have you had neck pain?” and “In the past month, have you had shoulder pain?” Responses were coded “Yes = 1” and “No = 0” to derive past-month neck pain and shoulder pain. NSP was defined as reporting neck pain and/or shoulder pain during the past month. Participants who reported pain in at least one of these regions were coded as having NSP (1), and those reporting no pain in either region were coded as without NSP (0).

#### 2.4.2 Physical activity (PA)

The Chinese version of the Physical Activity Questionnaire for Children (PAQ-CN) was used to evaluate children's PA levels. The questionnaire measures overall PA during the past 7 days for children aged 7–18 years (35). A five-point Likert response system (1–5) is applied to all nine items included in the instrument. PA was categorized as low (PAQ-CN ≤ 2.00), moderate (2.00 < PAQ-CN ≤ 3.00), or high (PAQ-CN > 3.00). The PAQ-CN demonstrated acceptable internal reliability (Cronbach's *α* = 0.82) and has been validated in studies conducted among Chinese school-aged populations (36).

#### 2.4.3 Fundamental motor skills (FMS)

The Test of Gross Motor Development, Third Edition (TGMD-3) was adopted to assess participants' FMS. FMS refers to proficiency in common FMS with specific patterns (37). FMS are categorized into two domains: six locomotor skills (e.g., running) and seven ball skills (e.g., swinging a racket with one hand). Two trials are completed for each skill, and their scores are summed to produce the final score. Locomotor skill scores range between 0 and 46, and ball skill scores range between 0 and 54.

#### 2.4.4 Psychological distress

We adapted the Chinese version of the Revised Child Anxiety and Depression Scale–25 (RCADS-25) to assess children's anxiety and depressive symptoms. The original questionnaire consists of 25 items, which form two subscales: 15 items for anxiety and 10 items for depression (38). The Chinese version retains the original two-factor structure and has been validated in multiple school-based samples in China.

In our preliminary analyses, item 18 of the RCADS-25 (“I think about death”) was considered inappropriate for primary school children due to its sensitive content and potential ethical concerns when administered in a school-based survey within the Chinese educational context. Therefore, this item was removed from the survey. After deletion, the anxiety subscale contained 14 items and the depression subscale 10 items, for a total of 24 items. The modified RCADS-24 demonstrated acceptable internal consistency (Cronbach's *α* = 0.709). A four-point Likert-type metric was applied to all items. The overall scale permits scores between 0 and 72, while the anxiety and depression domains allow ranges of 0–42 and 0–30. Elevated scores reflect greater symptom severity.

#### 2.4.5 Quality of life (QoL)

QoL was evaluated using the Chinese adaptation of the Pediatric Quality of Life Inventory (PedsQL). The questionnaire assesses four dimensions (39). Each item is completed on a five-point response scale. Following PedsQL scoring procedures, all items are reverse-coded and then converted to a 0–100 metric, with higher scores indicating better QoL.

### 2.5 Study procedure

Upon obtaining ethical approval from the Human Research Ethics Committee of Universiti Sains Malaysia (USM/JEPeM/PP/24060501) and permission from the principals of the selected schools, data collection was conducted by explaining the study procedures to the students in the presence of their respective teachers.

Before the commencement of data collection, written consent was provided by parents or legal guardians, and assent was granted by the participating children. The participation was voluntary. Data was collected from February to April 2025, with each assessment session requiring approximately 25 min for the TGMD-3 test and about 35 min to complete four self-administered questionnaires, including the PAQ-CN, NMQ, RCADS-25, and PedsQL.

### 2.6 Statistical analysis

All data were analyzed using IBM SPSS Statistics version 27. Descriptive statistics were applied to summarize the participants' characteristics and study variables. Means and standard deviations were calculated for numerical variables, while frequencies and percentages were used for categorical variables. Univariable and multivariable binary logistic regression were conducted to identify factors associated with NSP. Results were reported in terms of crude odds ratios (ORs) and adjusted odds ratios (AORs) with 95% confidence intervals (CIs). Multicollinearity among independent variables was assessed using variance inflation factors (VIF).

## 3 Results

### 3.1 Sociodemographic data

Out of the 370 questionnaires distributed, 362 (97.8%) were completed and returned. Participant demographic characteristics are shown in Table 1. Participants were on average 11.39 ± 0.83 years old. Of the 362 students, 173 (47.79%) were male and 189 (52.21%) were female.

**Table 1:** Demographic characteristics of participants (*n* = 362).

Variables			Frequencies (%)	Mean ± *SD*
Gender				
Male			173 (47.79)	
Female			189 (52.21)	
Age(years)				11.39 ± 0.83
Age group(years)				
10.0–10.9			133 (36.74)	
11.0–11.9			103 (28.45)	
12.0–12.9			126 (34.81)	
Region	School	Class^a^		
Changan District	1	5-1	45 (12.43)	
	2	6-3	46 (12.71)	
Xinhua District	3	5-2	43 (11.88)	
	4	6-2	47 (12.98)	
Yuhua District	5	5-3	44 (12.15)	
	6	6-1	45 (12.43)	
Qiaoxi District	7	5-1	46 (12.71)	
	8	6-1	46 (12.71)	

SD, standard deviation.

a Classes are divided in the form of Grade-Class, for example with 1–2 representing the second class of the first grade.

### 3.2 Distribution of participants' MSP among primary school students

The distribution of MSP frequency across different body parts for the past 1-month and 6-month periods is presented in Table 2. In the past 1-month period, neck pain was the most frequently reported site (27.90%), followed by the shoulder (16.85%) and lower back (14.64%). In comparison, there was an overall decrease in the frequency of pain reports during the 6-month review period, but the neck (15.75%), shoulder (8.84%), and lower back (6.91%) remained the most frequently reported areas.

**Table 2:** Distribution of participants’ MSP at 1-month and 6-month.

Variables	1-month	6-month
	Frequency (%)	Frequency (%)
Neck	101 (27.90)	57 (15.75)
Shoulder	61 (16.85)	32 (.84)
Elbow	14 (3.87)	3 (0.83)
Upper back	40 (11.05)	19 (5.25)
Lower back	53 (14.64)	25 (6.91)
Wrist/hand	24 (6.63)	3 (0.83)
Thigh	30 (8.29)	6 (1.66)
Knee	31 (8.56)	10 (2.76)
Ankle/foot	17 (4.70)	3 (0.83)

### 3.3 Factor associated with NSP

Table 3 presents the univariable findings examining the relationships between sociodemographic factors, PA, FMS, psychological distress, and QoL with NSP during the past month. A significant gender difference was observed in NSP prevalence (*p* = 0.022), with females reporting a higher prevalence (39.15%) than males (27.75%). Logistic regression further indicated that females had 1.68 times the odds of reporting NSP compared to males (OR = 1.68, 95% CI: 1.08–2.01, *p* = 0.022).

**Table 3:** Univariable analysis of sociodemographic, PA, FMS, psychological and QoL variables with 1-month NSP.

Variables	Total (%)/M ± SD	n (%)/M ± SD		*p*-value	Odds ratio (OR) (95% CI)
		Yes	No		
Gender					
Male	173 (47.80)	48 (27.75)	125 (72.25)		1
Female	189 (52.21)	74 (39.15)	115 (60.85)	0.022	1.68 (1.08–2.61)*
Age group(years)				0.114	
10.0–10.9	133 (36.74)	37 (27.82)	96 (72.18)		1
11.0–11.9	103 (28.45)	42 (40.78)	61 (59.22)		
12.0–12.9	126 (34.81)	43 (34.13)	83 (65.87)	0.273	1.34 (0.79–2.28)
BMI (kg/m^2^)	18.30 ± 3.53	18.71 ± 3.68	18.09 ± 3.44	0.119	1.05 (0.99–1.12)
PA level					
Low	139 (38.40)	55 (39.57)	84 (60.43)		1
Moderate	141 (38.95)	32 (22.70)	109 (77.30)	0.003	0.45 (0.27–0.76)**
High	82 (22.65)	35 (42.68)	47 (57.32)	0.649	1.14 (0.65–1.98)
FMS	71.01 ± 8.60	69.83 ± 8.08	71.61 ± 8.81	0.063	0.98 (0.95–1.00)
Ball skill	38.69 ± 6.27	37.86 ± 5.95	39.11 ± 6.40	0.075	0.97 (0.94–1.00)
Locomotor skill	32.28 ± 5.25	31.83 ± 5.22	32.52 ± 5.27	0.239	0.98 (0.94–1.02)
Psychological	13.67 ± 5.43	14.78 ± 5.98	13.10 ± 5.05	0.008	1.06 (1.02–1.10)**
Anxiety	7.24 ± 3.16	7.68 ± 3.38	6.96 ± 3.02	0.019	1.09 (1.01–1.16)*
Depression	6.42 ± 2.98	6.98 ± 3.13	6.14 ± 2.86	0.012	1.09 (1.02–1.18)*
QoL	76.76 ± 10.53	74.81 ± 11.54	77.75 ± 9.85	0.012	0.97 (0.95–0.99)*
Physical functioning	76.98 ± 13.29	74.41 ± 15.27	78.28 ± 11.99	0.015	0.98 (0.96–0.99)*
Psychosocial health	76.87 ± 11.11	75.48 ± 11.88	77.58 ± 10.66	0.088	0.98 (0.96–1.00)
Emotional functioning	77.97 ± 13.81	76.60 ± 13.55	78.67 ± 13.92	0.178	0.99 (0.97–1.00)
Social functioning	78.67 ± 14.14	76.60 ± 15.23	79.73 ± 13.47	0.046	0.98 (0.97–1.00)
School functioning	76.10 ± 15.60	74.30 ± 15.84	77.02 ± 15.44	0.117	0.99 (0.98–1.00)

CI, confidence interval; OR, odds ratio; SD, standard deviation.

* *P* < 0.05.

** *P* < 0.01.

PA level was significantly associated with the prevalence of NSP. In the moderate PA group, the reported rate of NSP was 22.70%, which was considerably lower than in the low-level group (39.57%, *p* = 0.003), and logistic regression analysis showed that moderate PA level was associated with lower odds of NSP (OR = 0.45, 95% CI: 0.27–0.76, *p* = 0.003).

For the psychological distress, in univariable logistic regression, each one-point increase in psychological symptoms was linked to higher odds of NSP (OR = 1.06, 95% CI 1.02–1.10; *p* = 0.008). Anxiety symptoms were slightly higher in the NSP group and showed a positive association with NSP (OR = 1.09, 95% CI 1.01–1.16, *p* = 0.019), whereas depression was clearly higher in the NSP group and was independently related to NSP (OR = 1.09, 95% CI 1.01–1.16, *p* = 0.012).

For QoL, a higher score was protective: each one-point increase in the total score was associated with lower prevalence of NSP (OR = 0.97, 95% CI 0.95–0.99, *p* = 0.012). At the domain level, physical functioning was reduced in the NSP group and was inversely related to NSP (OR = 0.98, 95% CI 0.96–0.99, *p* = 0.015).

Prior to multivariable logistic regression, multicollinearity was assessed, and no evidence of multicollinearity was detected. All VIF values ranged from 1.009 to 1.208, well below the recommended threshold of 5 (40). Multivariable analysis is shown in Table 4. Females had higher odds of reporting NSP than males (AOR=1.61, 95% CI 1.01–2.56, *p* = 0.047). Compared with the low-PA group, the moderate-PA group had significantly lower odds of NSP (AOR=0.51, 95% CI 0.29–0.89, *p* = 0.018). For each one-point increase in the psychological score, the odds of NSP increased by 4% (AOR=1.04, 95% CI 1.01–1.09, *p* = 0.036). For each one-point increase in QoL, the odds of NSP decreased by 2% (AOR=0.98, 95% CI 0.95–0.99, *p* = 0.026). Overall, female gender and poorer psychological status were independently associated with higher odds of NSP, whereas moderate PA and higher QoL were associated with lower odds of NSP. No additional benefit was observed for high PA, suggesting a potential non-linear association between PA and NSP.

**Table 4:** Multivariable analysis of variables associated with 1-month NSP.

Variable	B	SE	*p*-value	Odds ratio (OR) (95% CI)
Gender (ref: Male)				
Female	0.474	0.238	0.047	1.61 (1.01–2.56)*
PA level (ref: Low)				
Middle	−0.669	0.283	0.018	0.51 (0.29–0.89)*
High	0.508	0.327	0.121	1.66 (0.88–3.16)
BMI	0.051	0.032	0.119	1.05 (0.99–1.12)
FMS	−0.029	0.015	0.053	0.97 (0.94–1.00)
Psychological	0.046	0.022	0.036	1.04 (1.01–1.09)
Quality of life	−0.026	0.011	0.026	0.98 (0.95–0.99)*

CI, confidence interval; AOR, adjusted odds ratio; SE, standard error; B, regression coefficient.

* *P* < 0.05.

## 4 Discussion

This is the first study to investigate the prevalence of NSP and its associated factors among primary school children in China. We found a one-month prevalence of NSP (33.7%), with the neck (27.9%), shoulder (16.9%), and lower back (14.6%) being the most frequently affected regions. The present findings align with earlier research. For instance, an Iranian study reported that 32.3% of primary school students experienced NSP symptoms (6), and a study conducted in Finland found that 32% of children reported NSP at least once weekly (4). These findings suggest that NSP remains prevalent among primary school children and may represent a significant public health concern, highlighting the importance of early school-based preventive strategies.

Our findings are consistent with earlier studies that reported a significantly higher odds of NSP among females than males (4, 6, 7, 41). Several hypotheses may account for this disparity. First, males tend to be more enthusiastic about sports than females and engaging in suitable exercise may facilitate muscle relaxation and enhanced strength in the neck and shoulder region, thereby alleviating NSP symptoms (41). Second, previous studies have shown that females generally exhibit lower pain thresholds and reduced pain tolerance, making them more susceptible to experiencing greater discomfort (42). Third, most studies have confirmed that sex disparities exist in pain perception, with females tending to exhibit increased sensitivity and a higher likelihood of clinical pain (43). The observed gender difference in NSP suggests that school-based prevention programs may benefit from a gender-sensitive approach within a biopsychosocial framework. Interventions may consider differences in PA patterns, pain perception, and psychosocial factors between boys and girls. However, these strategies should be applied carefully and consider individual differences, rather than making assumptions based solely on gender. Future longitudinal and intervention studies are needed to determine whether tailored approaches provide additional benefit over universal programs.

In this study, moderate PA was associated with lower odds of NSP compared with low PA, consistent with findings reported by Guddal in Norwegian adolescents (44). The association may partly reflect that physically active adolescents generally have healthier lifestyles and better overall health (e.g., reduced prolonged sitting and greater muscle endurance), which may be associated with lower odds of NSP (44). These findings underscore the importance of PA intensity, although different types of exercise may exert their effects through distinct physiological pathways. Evidence from a randomized trial in trapezius myalgia indicates that resistance training and aerobic training alleviate pain via distinct pathways; progressive resistance work induces hypertrophy of type II fibers and increases satellite-cell content, whereas aerobic training produces session-by-session analgesia, enhances muscle perfusion or oxygenation during standardized work tasks, and reduces peripheral and central pain sensitivity (45). These adaptations plausibly lower mechanical load and nociceptive drive in the cervical–shoulder region, providing a mechanistic rationale for the protective association observed with moderate-intensity PA. Interestingly, we observed a non-significant trend toward higher odds of NSP in the high-PA group. This pattern may indicate a possible non-linear relationship between PA and NSP; however, this observation is exploratory and was not formally tested in the present study. A prospective Danish study reported comparable findings and found that transitioning from inactivity to moderate PA was associated with a lower likelihood of spinal pain, whereas vigorous PA was associated with a higher likelihood of spinal pain (46). However, the observed association between PA and NSP may be subject to reverse causation, as children experiencing pain may have lower activity levels. Given the cross-sectional design of this study, causal inferences cannot be established.

Previous evidence has suggested several biological and psychological pathways that may be relevant to the association between PA and NSP. First, studies have shown that NSP may induce structural and functional changes in the brain, such as reductions in the volume and density of gray and white matter (47, 48), as well as alterations in neural connectivity across brain regions (49). The insular cortex, a key region involved in pain processing, has been found to show activity levels closely associated with pain intensity; both short- and long-term exercise interventions can modulate this activity (50). In addition, descending inhibitory pathways and endogenous analgesic systems are activated during exercise, contributing to reduced pain perception (51). Moreover, PA plays an important physiological regulatory role by reducing inflammatory cytokine levels, improving joint flexibility, and enhancing the body's ability to adapt to physical stress (47). Notedly, on the psychological level, PA contributes to alleviating symptoms of anxiety and depression and enhancing well-being through mechanisms such as increasing life satisfaction, reducing stress hormones (e.g., cortisol), and stimulating endorphin release (52). Future studies should examine how these mechanisms interact across multiple levels to better understand the association between PA and NSP.

Our study indicates that psychological distress was independently associated with NSP. On the one hand, elevated levels of anxiety were significantly associated with NSP in children. Longitudinal evidence suggests that anxiety in early adolescence predicts increased pain-related anxiety and functional impairment in later adolescence (53). However, the existing evidence remains inconsistent. For example, among 17 studies, only 10 reported a higher likelihood of neck pain among individuals with anxiety disorders (54). These discrepancies may be attributable to differences in measurement instruments, cultural contexts, and study designs. On the other hand, among adolescents, higher depressive symptom scores have been identified as one of the strongest factors associated with NSP. In addition, 9.6% of Norwegian adolescents exhibited concurrent NSP and depressive symptoms (55). These findings suggest a potential bidirectional relationship between pain and depression, whereby affective disturbances may alter pain processing, while nociceptive stimulation may in turn precipitate or exacerbate depressive states (56).

In addition, previous evidence has suggested several bio-psychological pathways that may be relevant to the association among anxiety, depression, and pain perception. Functional neuroimaging studies suggest altered engagement and connectivity of insular and prefrontal regions in individuals with anxiety and depressive symptoms, implicating disrupted affective processing and prefrontal control mechanisms relevant to pain modulation (57, 58). Concurrently, autonomic nervous system dysregulation, hyperactivity of the hypothalamic–pituitary–adrenal axis, and elevated inflammatory responses observed in individuals with anxiety and depression may contribute to heightened pain sensitivity (59, 60). Moreover, neurotransmitters such as norepinephrine and serotonin, which play central roles in both emotional regulation and descending pain modulation, may, when dysregulated, reduce the body's capacity to regulate nociceptive signaling, thereby amplifying the subjective experience of pain (61, 62). Notably, Chinese students are exposed to substantial academic pressure, with high expectations for performance across multiple domains, which may be related to psychological distress. Therefore, future school-based intervention strategies should incorporate mental health components within a comprehensive preventive framework.

We further found that higher QoL scores were associated with lower odds of NSP in children. However, it should be noted that the effect size was small (AOR ≈ 0.98), and thus the clinical relevance of this association should be interpreted with caution. Previous studies have consistently demonstrated that MSP in children is significantly associated with lower QoL (63, 64). Longitudinal evidence further indicates that adolescents who reported any MSP at age 13 exhibited significantly lower physical health-related QoL scores at age 18 compared with their peers (63). Nevertheless, although prior research has explored the association between childhood pain and QoL, systematic analyses focusing on specific pain sites (e.g., NSP) and differential effects across QoL domains remain limited. The present study further revealed significant differences in the physical functioning domain between children with and without NSP. This difference may be related to pain-related activity limitations and increased discomfort, which could impair children's functional performance and tolerance in both PA and classroom learning contexts (65). This study is among the few to examine the association between NSP and QoL among Chinese primary school students. Overall, our findings suggest that NSP may be associated with poorer overall QoL, particularly in the physical functioning domain. These results underscore the importance of early identification and intervention strategies within school and community settings.

Notably, FMS was not significantly associated with NSP in this study. Previous research has suggested that poorer motor skills in early childhood, particularly deficits in fine motor skills and coordination, may be associated with a higher likelihood of severe neck pain (66). These findings suggest that motor skill development in early childhood may be related to NSP later in adolescence or adulthood. Therefore, although no association between FMS and NSP was observed in the present study, potential indirect or long-term associations warrant further investigation in longitudinal research designs.

The strengths of this study are threefold. First, it is the first to focus on Chinese primary school children, addressing the evidence gap on the prevalence and associated factors of NSP in this population. Second, the use of a one-month recall period likely minimized recall bias. Third, this study adopted a biopsychosocial framework by simultaneously examining biological, psychological, and social factors. This multidimensional perspective provides a more comprehensive understanding of NSP in primary school children. However, this study has several limitations. Given the cross-sectional design of this study, causal relationships between NSP and its associated factors cannot be established. Therefore, future longitudinal studies are needed to further examine these relationships. Furthermore, several important confounding factors were not included in the regression models, such as screen time, sitting posture, school workload, and sleep duration. The absence of these variables may have introduced residual confounding into the estimated associations between NSP and the studied factors. As these factors may be related to both the exposures and NSP, the observed associations may have been overestimated or underestimated. For example, prolonged screen time and poor sitting posture may be associated with both lower PA participation and a higher likelihood of reporting NSP (7), while insufficient sleep and heavy academic workload may be related to psychological distress, poorer QoL, and NSP (4, 6). Therefore, the associations observed in this study should be interpreted with caution. Future studies should incorporate these factors and adopt more comprehensive analytical models to further examine these associations. In addition, NSP was assessed using a binary self-report measure, which did not capture pain intensity, duration, or frequency. This measurement approach may have limited the clinical interpretability of the findings because it could not distinguish between mild, transient discomfort and more persistent or clinically meaningful pain. Therefore, the absence of detailed pain characteristics may have led to potential misclassification bias, and the prevalence estimates should be interpreted with caution. Furthermore, potential misclassification of NSP status may have affected the accuracy of the regression estimates and may have attenuated the observed associations between NSP and its associated factors. Future studies are warranted to incorporate more detailed pain assessments, including pain intensity, duration, and frequency, to improve the clinical interpretation of pediatric NSP and reduce the potential for misclassification bias.

## 5 Conclusion

This is the first study in China to investigate the prevalence and associated factors of NSP among primary school children. Moderate-intensity PA is associated with lower NSP prevalence, while lower QoL and poorer psychological status are associated with higher odds of reporting NSP. Early, school-based programs that integrate PA promotion with psychological support are warranted, and longitudinal research should verify these relationships.

## Data Availability

The raw data supporting the conclusions of this article will be made available by the authors, without undue reservation.
